# The role of mechanical control of biofilm in the salivary pH after sucrose exposure in children with early childhood caries

**DOI:** 10.1038/s41598-021-86861-4

**Published:** 2021-04-05

**Authors:** Aline Tavares Lima-Holanda, Emerson Tavares de Sousa, Marinês Nobre-dos-Santos, Carolina Steiner-Oliveira

**Affiliations:** Department of Pediatric Dentistry, Piracicaba Dental School, University of Campinas-UNICAMP, Limeira Avenue 901, Piracicaba, SP CEP 13414-903 Brazil

**Keywords:** Dentistry, Dental caries

## Abstract

This quasi-experimental study sought to investigate if the mechanical control of biofilm (3-times-a-day) modifies the saliva’s ability to buffer the oral environment after 20% sucrose rinse (SR_20%_) in children with early childhood caries (ECC). Here, SR_20%_ reduced the saliva’s pH in both groups and the mechanical control of biofilm had a greater effect on this parameter after SR_20%_ in CF children. The mechanical control of biofilm evidenced a higher buffering capacity in CF children before SR_20%_, which was not observed after SR_20%_. Otherwise, the absence of mechanical control of biofilm showed that buffering capacity was comparable in the two groups before SR_20%_, whereas after SR_20%_ the saliva’s buffering capacity of CF children was higher than ECC children. When biofilm was mechanically controlled, carbonic anhydrase VI activity did not change after SR_20%_ whereas the absence of mechanical control of biofilm reduced this enzyme activity after SR_20%_. In conclusion, the mechanical control of biofilm did not change saliva’s ability to buffer the oral environment after SR_20%_ in children with ECC. On the other hand, CF children appeared to regulate more effectively the saliva’s pH than ECC children while the absence of mechanical control of biofilm mediated their pH-modifying ability after SR_20%_.

## Introduction

The dental biofilm is an essential organized structure in oral health due to the symbiotic relationship between commensal bacteria and the host^1^. Its biological function and architecture are determined by saliva composition and properties, and most importantly, by the dominant nutritional source in the child's diet^2^. Accordingly, if the dominant nutritional source shifts toward a frequent sucrose exposure, an adaptive metabolism provides a coercive environment for a dysbiotic state^3^. In this sense, a highly specialized ecosystem with important modifications in the physical–chemical properties of saliva can be expected^4^.

As early demonstrated, sucrose exposure changes the salivary electrolytic behavior (calcium, phosphate, and fluoride)^5^ and saliva enzymes as carbonic anhydrase VI^6^, which may harmful to the salivary buffering capacity since a higher pH fall after sucrose exposure was found in the saliva of children with ECC. Thus, we may speculate that ECC (as a chronic process) can disrupt the oral ecosystem by a mechanism linked with intraoral pH control, possibly weakening the saliva's ability to buffer the medium after a cariogenic challenge.

The saliva buffering capacity works more efficiently during stimulated flow rates^7^ due to the increase in the bicarbonate (${HCO}_{3}^{-}$) secretion and loss of CO_2_ that will drive the equilibrium of bicarbonate system equation to the left (more alkaline) direction as represented here: [CO_2_ + H_2_O ⇄ H_2_CO_3_ ⇄ ${HCO}_{3}^{-}$  + H^+^ ]^8^. This phenomenon is upregulated by the catalytic property of carbonic anhydrase VI, which accelerates the neutralization of the chemical aggression caused by the microbial sucrose metabolism^9–11^. Under the limits of an ideal scenario, this biochemical model works well and may represent a physiological condition; however, the complex interactions between saliva and biofilm, under influence of dental caries, make the understanding of this buffer system rather complicated.

Dental caries is a disease that promotes changes in the expression and catalytic activity of the CA VI in saliva^12^ and biofilm^13^, and this process may be related to the likelihood of caries lesions development in children^14^. Some studies evidenced that higher enzyme activity was linked to a lower salivary buffering capacity in schoolchildren with caries experience^6,12,14^. The research conducted by Frasseto et al.^15^ showed that after a 20% sucrose rinse, a different enzymatic behavior was observed when caries-free children were compared with children with ECC. This finding suggests that the enzymatic behavior of CA VI and it`s ability to catalyze the saliva neutralization after a cariogenic challenge was influenced by ECC. Nevertheless, since no information regarding the saliva pH and buffering capacity was provided by these authors, it is difficult to deduce the impact that the CA VI activity would have on these physiological parameters. Given the importance of α-amylase (α-AML) on oral physiology and dental caries dynamic^16,17^ as well as its biochemical link to CA VI^18,19^ and biological function^10,20,21^ in the oral cavity, we hypothesized a contributory mechanism for pH homeostasis in the oral environment.

In addition to this background, we must highlight that one of the primary preventive strategies to manage early childhood caries is to provide caregivers effective strategies to properly control biofilm accumulation^22^. However, despite the strong effect of this intervention on ECC^23^, its impact on the oral environment is not fully understood. Therefore, this study aimed at investigating, in a short-term intervention, if the mechanical control of biofilm can change the saliva ability to buffer the oral environment after sucrose exposure in children with early childhood caries.

## Results

Table 1 shows the sample characteristics according to dental caries status. The sex ratio in each group was similar. Sucrose ingestion (based on frequency, amount, and % in diet), was higher in the ECC group when compared with the CF group.

**Table 1:** Sample characteristics of volunteers.

	CF	ECC	α (Effect Size)
dmfs plus IL^¥^—Median (IQR)	0	7.00 (10.00)	*
Sex (M:F)	1.15:1.00	1.00:1.00	0.79
Total sugar amount (g)—Mean (SD)	287.25 (41.16)	306.50 (30.80)	0.026 (0.52)
Total sugar frequency (times/day)—Mean (SD)	5.18 (1.55)	5.86 (1.08)	0.032 (0.50)
Percentage of sugar in diet (%)^$^—Mean (SD)	53.46 (3.79)	55.09 (3.27)	0.046 (0.45)

* Result not computed.

Continuous outcomes were tested using an independent t-test for α and Hedges’ g for effect size. Sex distribution across the groups was tested using Chi-squared.

^¥^The decomposed index was: 60% of WSL, 20% of active cavitated lesion, 11% of filled without decay, 7% of chronic cavitated lesion, and 2% of filled with chronic lesion.

^$^The calculation was based on the total sugar amount concerning all macronutrients from the diet. *dmfs *number of decayed, missing, or filled surfaces; ACL – active caries lesions.

**Figure 1:**
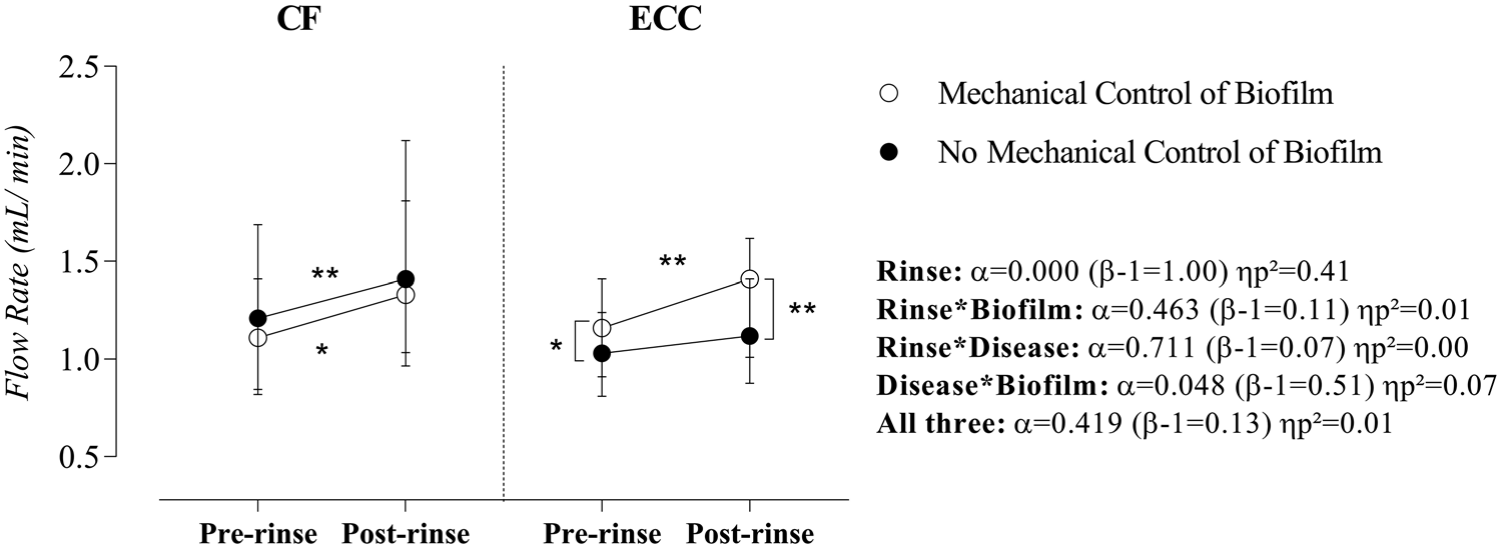
Effect of rinse, mechanical control of biofilm, and disease on salivary flow rate. Statistical analyses were performed with a sample of 56 volunteers, 28 per group. ηp^2^: Partial eta squared. Data plotted as median and interquartile range (due to non-Gaussian distribution). To fulfill the ANOVA premises, we used the log10 transformation. Considering the presence of significant interactions, the main effects of the disease and biofilm were suppressed. A single asterisk represents a significant p-value ≤ 0.05. A double asterisk represents a p-value ≤ 0.01. Simple effects for Rinse in CF children—Mechanical control of biofilm: α = 0.01 (β-1 = 0.75) ηp^2^ = 0.12 and No mechanical control of biofilm: α = 0.003 (β-1 = 0.86) ηp^2^ = 0.15). Simple effects for Rinse in children with ECC—Mechanical control of biofilm: α = 0.002 (β-1 = 0.88) ηp^2^ = 0.16 and No mechanical control of biofilm: α = 0.08 (β-1 = 0.42) ηp^2^ = 0.06). Simple effects for Biofilm in CF children—Pre-rinse: α = 0.72 (β-1 = 0.07) ηp^2^ = 0.002 and Post-rinse: α = 0.78 (β-1 = 0.06) ηp^2^ = 0.002. Simple effects for Biofilm in children with ECC—Pre-rinse: α = 0.02 (β-1 = 0.69) ηp^2^ = 0.103 and Post-rinse: α = 0.002 (β-1 = 0.88) ηp^2^ = 0.16. Simple Effects for Disease: No significant effect considering a p-value > 0.05. Supplementary material provides complementary information regarding data statistics.

Salivary flow rate increased after sucrose rinse (α = 0.000, β = 1.00, ηp^2^ = 0.41) and this effect was independent of the disease and biofilm. Since a significant interaction between disease and biofilm (α = 0.048, β = 0.51, ηp^2^ = 0.07) was evidenced, the simple main effect demonstrated that the mechanical control of biofilm raises SFR in children with ECC (pre-rinse: α = 0.016, β = 0.69, ηp^2^ = 0.10, and post-rinse: α = 0.002, β = 0.88, ηp^2^ = 0.16), as shown in Fig. 1.

**Figure 2:**
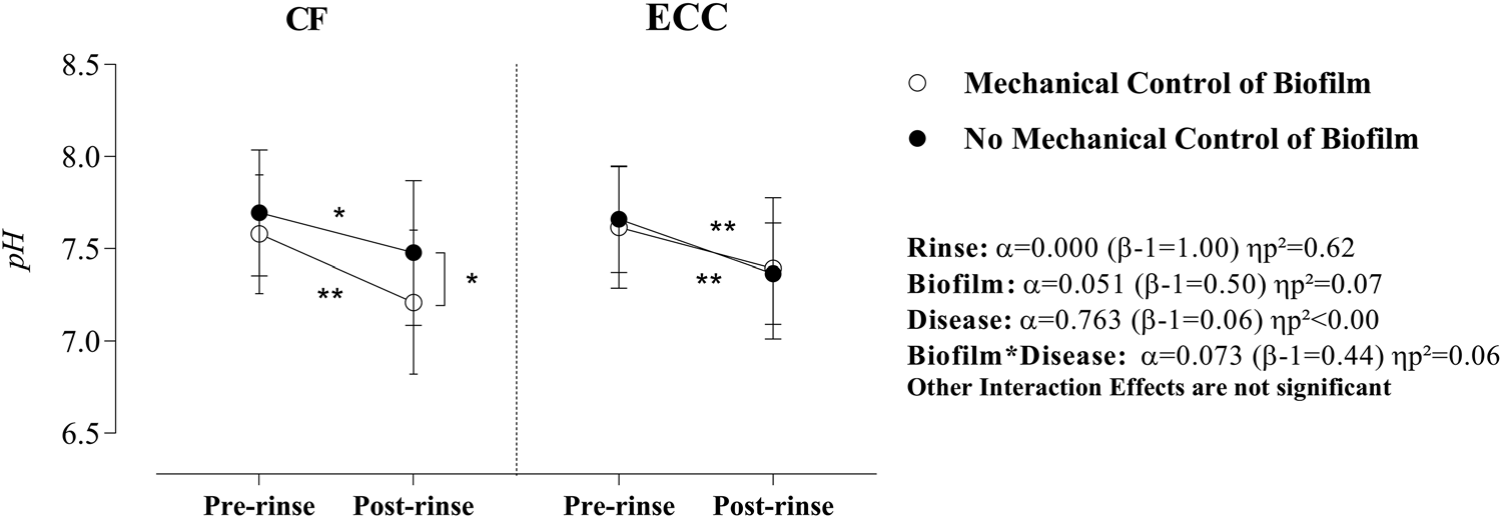
Effect of rinse, mechanical control of biofilm, and disease on saliva pH. Statistical analyses were performed in a sample of 56 volunteers, 28 per group. ηp^2^: Partial eta squared. Data plotted as mean and standard deviations (due to Gaussian distribution). Non-significant interactions were suppressed. A single asterisk represents a significant p-value ≤ 0.05. A double asterisk represents p-value ≤ 0.01. Simple effects for Rinse in CF children—Mechanical control of biofilm: α = 0.000 (β-1 = 1.00) ηp^2^ = 0.45 and No mechanical control of biofilm: α = 0.005 (β-1 = 0.82) ηp^2^ = 0.14). Simple effects for Rinse in children with ECC—Mechanical control of biofilm: α = 0.000 (β-1 = 0.97) ηp^2^ = 0.23 and No mechanical control of biofilm: α = 0.000 (β-1 = 0.97) ηp^2^ = 0.23. Simple effects for Biofilm in CF children—Pre-rinse: α = 0.12 (β-1 = 0.35) ηp^2^ = 0.05 and Post-rinse: α = 0.01 (β-1 = 0.75) ηp^2^ = 0.12. Simple effects for Biofilm in children with ECC—Pre-rinse: α = 0.54 (β-1 = 0.09) ηp^2^ = 0.007 and Post-rinse: α = 0.78 (β-1 = 0.06) ηp^2^ = 0.001. Simple Effects for Disease: No significant effect considering a p-value > 0.05. Supplementary material provides complementary information regarding data statistics.

Sucrose rinse promoted a large (high ηp^2^) and significant effect on the saliva pH, which was not affected by biofilm or disease effects (no significant interaction). Moreover, in CF children and after the sucrose rinse, there was a significant difference (p = 0.017, β = 0.75, ηp^2^ = 0.01) in saliva pH when the mechanical control of biofilm situation was compared with no mechanical control of biofilm (Fig. 2).

**Figure 3:**
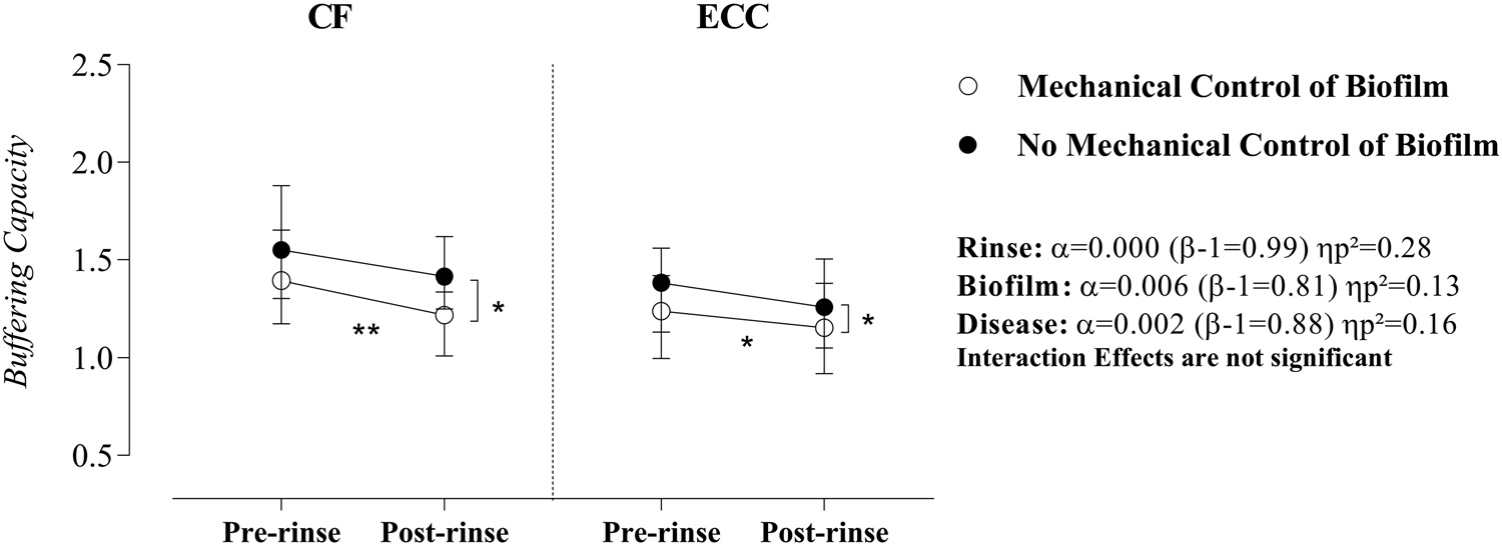
Effect of rinse, mechanical control of biofilm, and disease on saliva buffering capacity. Statistical analyses were performed with a sample of 56 volunteers, 28 per group. ηp^2^: Partial eta squared. Data plotted as median and interquartile range (due to non-Gaussian distribution). To fulfill the ANOVA premises, we used the log10 transformation. Non-significant interactions were suppressed. A single asterisk represents a significant p-value ≤ 0.05. A double asterisk represents p-value ≤ 0.01. Simple effects for Rinse in CF children—Mechanical control of biofilm: α = 0.007 (β-1 = 0.79) ηp^2^ = 0.13 and No mechanical control of biofilm: α = 0.23 (β-1 = 0.22) ηp^2^ = 0.03). Simple effects for Rinse in children with ECC—Mechanical control of biofilm: α = 0.01 (β-1 = 0.72) ηp^2^ = 0.11 and No mechanical control of biofilm: α = 0.07 (β-1 = 0.43) ηp^2^ = 0.06. Simple effects for Biofilm in CF children—Pre-rinse: α = 0.19 (β-1 = 0.26) ηp^2^ = 0.03 and Post-rinse: α = 0.04 (β-1 = 0.55) ηp^2^ = 0.08. Simple effects for Biofilm in children with ECC—Pre-rinse: α = 0.09 (β-1 = 0.39) ηp^2^ = 0.05 and Post-rinse: α = 0.04 (β-1 = 0.49) ηp^2^ = 0.07. Simple Effects for Disease in Pre-rinse condition—Mechanical control of biofilm: α = 0.04 (β-1 = 0.56) ηp^2^ = 0.08 and No mechanical control of biofilm: α = 0.12 (β-1 = 0.33) ηp^2^ = 0.04. Simple Effects for Disease in Post-rinse condition—Mechanical control of biofilm: α = 0.06 (β-1 = 0.46) ηp^2^ = 0.06 and No mechanical control of biofilm: α = 0.04 (β-1 = 0.56) ηp^2^ = 0.08. Supplementary material provides complementary information regarding data statistics.

The main effects evidenced that sucrose rinse, biofilm accumulation, and disease status had a strong and significant influence on the buffering capacity. Specifically, when the mechanical control of biofilm was performed, the difference between CF children and children with ECC became meaningful at the pre-rinse moment. However, no difference between the two groups was observed at the post-rinse moment. In contrast, when the biofilm was not disrupted, the buffering capacity was comparable in the two groups at the pre-rinse moment; but was greater in CF children after sucrose rinse. Additionally, we observed a greater reduction in the buffering capacity after sucrose rinse, when biofilm was controlled. The simple effect for biofilm evidenced that differences were statistically evident only at the post-rinse moment (CF: α = 0.04, β = 0.55, ηp^2^ = 0.08, and ECC: α = 0.05, = 0.50, ηp^2^ = 0.07). Regardless of the disease factor, sucrose rinse did not induce meaningful changes in the buffering capacity when the mechanical control of biofilm strategy was absent (Fig. 3).

**Figure 4:**
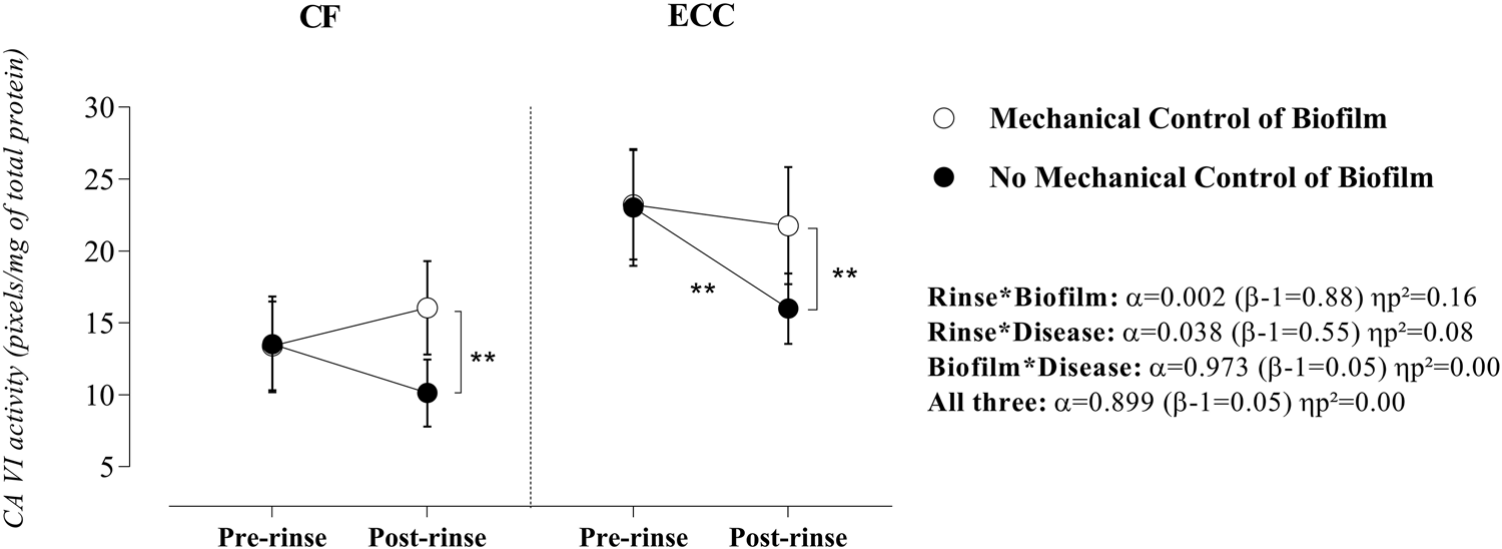
Effect of rinse, mechanical control of biofilm, and disease on salivary CA VI activity. Statistical analyses were performed with a sample of 56 volunteers, 28 per group. ηp^2^: Partial eta squared. Data plotted as mean and standard deviations (due to Gaussian distribution). Considering the presence of significant interactions, the main effects of rinse, disease, and biofilm were suppressed. A single asterisk represents a significant p-value ≤ 0.05. A double asterisk represents a p-value ≤ 0.01. Simple effects for Rinse in CF children—Mechanical control of biofilm: α = 0.15 (β-1 = 0.30) ηp^2^ = 0.04 and No mechanical control of biofilm: α = 0.07 (β-1 = 0.43) ηp^2^ = 0.06). Simple effects for Rinse in children with ECC—Mechanical control of biofilm: α = 0.42 (β-1 = 0.13) ηp^2^ = 0.01 and No mechanical control of biofilm: α = 0.000 (β-1 = 0.96) ηp^2^ = 0.21. Simple effects for Biofilm in CF children—Pre-rinse: α = 0.96 (β-1 = 0.05) ηp^2^ = 0.000 and Post-rinse: α = 0.003 (β-1 = 0.86) ηp^2^ = 0.15. Simple effects for Biofilm in children with ECC—Pre-rinse: α = 0.93 (β-1 = 0.05) ηp^2^ = 0.000 and Post-rinse: α = 0.004 (β-1 = 0.84) ηp^2^ = 0.14. Simple Effects for Disease in Pre-rinse condition—Mechanical control of biofilm: α = 0.000 (β-1 = 0.98) ηp^2^ = 0.24 and No mechanical control of biofilm: α = 0.000 (β-1 = 0.95) ηp^2^ = 0.20. Simple Effects for Disease in Post-rinse condition—Mechanical control of biofilm: α = 0.03 (β-1 = 0.60) ηp^2^ = 0.09 and No mechanical control of biofilm: α = 0.000 (β-1 = 0.94) ηp^2^ = 0.19. Supplementary material provides complementary information regarding data statistics.

The responsiveness of CA VI activity after sucrose rinse was largely influenced by both biofilm and disease (significant interaction). Particularly, the simple main effects demonstrated that, while the mechanical control of biofilm stabilized CA VI activity irrespective of the pre- and post-rinse comparisons (CF: p = 0.145, β = 0.31, ηp^2^ = 0.04, and ECC: p = 0.429, β = 0.13, ηp^2^ = 0.01), the abstention of the mechanical control of biofilm, induced a significant reduction in the CA VI activity after sucrose rinse (CF: α = 0.073, β = 0.44, ηp^2^ = 0.06 and ECC: α < 0.001, β = 0.96, ηp^2^ = 0.21) (Fig. 4).

**Figure 5:**
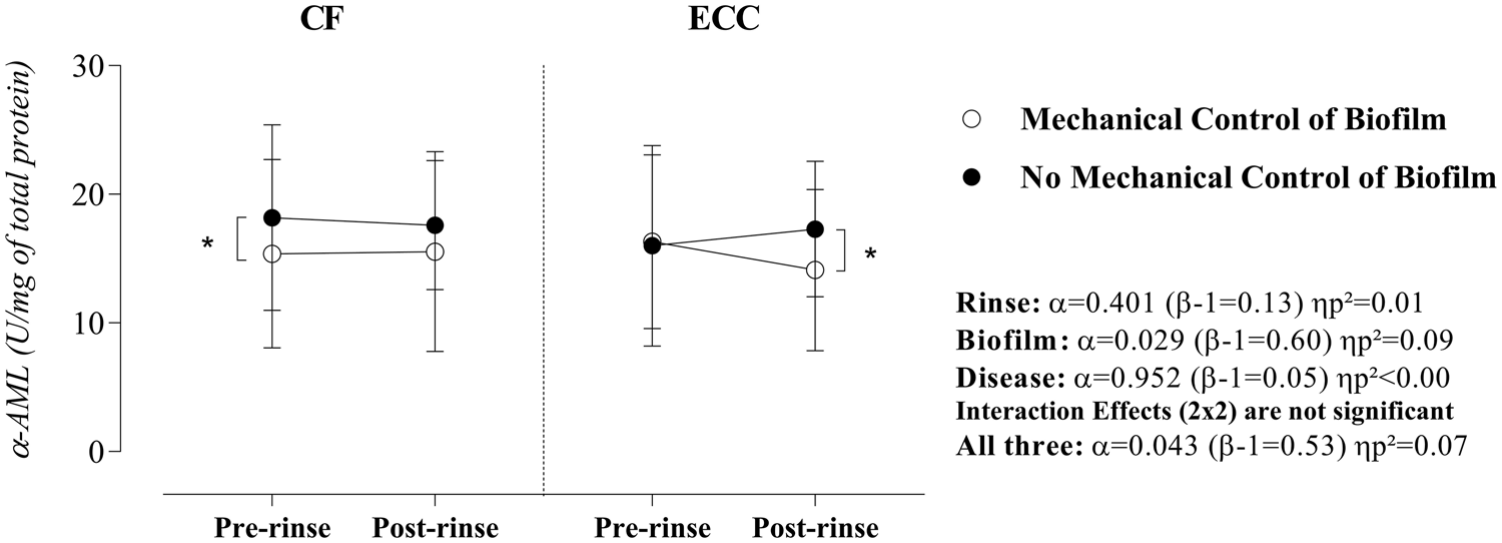
Effect of rinse, mechanical control of biofilm, and disease on salivary α-amylase. Statistical analyses were performed with a sample of 56 volunteers, 28 per group. ηp^2^: Partial eta squared. Data plotted as median and interquartile range (due to non-Gaussian distribution). To fulfill the ANOVA premises, we used the square root for data. Non-significant interactions were suppressed. A single asterisk represents a significant p-value ≤ 0.05. Simple effects for Rinse in CF children—Mechanical control of biofilm: α = 0.45 (β-1 = 0.12) ηp^2^ = 0.01 and No mechanical control of biofilm: α = 0.74 (β-1 = 0.06) ηp^2^ = 0.002. Simple effects for Rinse in children with ECC—Mechanical control of biofilm: α = 0.28 (β-1 = 0.19) ηp^2^ = 0.02 and No mechanical control of biofilm: α = 0.03 (β-1 = 0.59) ηp^2^ = 0.09. Simple effects for Biofilm in CF children—Pre-rinse: α = 0.04 (β-1 = 0.56) ηp^2^ = 0.08 and Post-rinse: α = 0.09 (β-1 = 0.40) ηp^2^ = 0.05. Simple effects for Biofilm in children with ECC—Pre-rinse: α = 0.42 (β-1 = 0.13) ηp^2^ = 0.01 and Post-rinse: α = 0.04 (β-1 = 0.56) ηp^2^ = 0.08. Simple Effects for Disease: No significant effect considering a p-value > 0.05. Supplementary material provides complementary information regarding data statistics.

The combined effect of sucrose rinse, disease, and the mechanical control of biofilm on the α-AML activity was observed (α = 0.043, β = 0.53, and ηp^2^ = 0.07). Therefore, we found a significant effect of biofilm at the pre-rinse moment (for CF children: p = 0.04, β = 0.56, and ηp^2^ = 0.08) and at the post-rinse moment (for children with ECC: p = 0.04, β = 0.56, and ηp^2^ = 0.08), as shown in Fig. 5.

To complement these results, supplementary material may provide detailed information regarding data statistics.

## Discussion

To reduce the sample heterogeneity and to make the proximal factors of caries disease more evident, this study was accomplished by using a sample of preschoolers with a narrow age range (between 4 and 5 years), similar socioeconomic status, access to 1100 ppm fluoride dentifrice, and 0.7 ppm fluoridated water, and who shared 5 meals per day between 7 a.m. and 5 p.m. (breakfast, a.m. snack, lunch, p.m. snack, and dinner). The comprehensive exploration of their diet, particularly fermentable carbohydrates intake, showed that the total amount (in grams) and frequency of sugar intake tend to be higher in children with ECC, probably due to their eating behavior at home. In addition, the group of children with ECC had high rates of active caries lesions (60% white spot lesion, and 20% of active cavitated lesion). Taken together, more than emphasizing the overwhelming influence of sugar in the caries process, these findings provide a reliable model to access the biological aspects of the “ecological catastrophe” in the oral cavity and warrant a comprehensive understanding of the boundaries of a healthy or disease state.

The variations in the oral environment’s pH generate the need for a regulatory buffering system, which is mostly provided by the bicarbonate buffer system when there is stimuli for saliva secretion (as reproduced in our study: chewing plus sweet stimuli)^8^. Given this context, considering the differences between CF children and children with ECC, we firstly evidenced that the mechanical control of biofilm had a significant impact on the saliva ability to neutralize the oral environment after sucrose exposure. This finding may reflect the transient shift of the oral ecosystem after sucrose exposure, thereby providing complementary evidence of the importance of good oral hygiene for the oral environment into the boundaries between healthy and diseased states.

In addition to the expected increase in salivary flow after sucrose rinse, the mechanical control of biofilm in children with ECC significantly increased the rate of saliva secretion and, more importantly, evoked a more intense response to the sweet stimuli (post-rinse moment). A possible explanation for this behavior could be grounded by an integrated mechanism related to the sucrose clearance time and the sweet taste perception threshold. The classical physiological model recognizes that the increase in the saliva flow rate after sucrose exposure is controlled by the sucrose dilution (clearance) as a negative feedback to maintain homeostasis^2,24^. In this context, the negative correlation between sweet taste perception threshold and carbohydrate intake^25^ and the longer oral clearance times in caries-affected children^26^ may be an adaptation of the taste threshold for sugar. In other words, the delay in sucrose clearance provides an increase in the gustatory threshold for sugar to control frequent salivary flow stimuli due to a higher habitual intake of sweet food. Thus, considering the dental practice scenario, we argue that proper oral hygiene in children with ECC overcomes the more adapted sweet taste perception due to the high sugar intake. Of importance, when the biofilm was not controlled, sucrose rinse did not change the SFR in children with ECC. Long-term strategies to limit sugar intake and mechanical control of biofilm can provide new insights into the influence of sweet taste perception and saliva clearance in dental caries susceptibility.

The sucrose rinse caused a significant reduction in the saliva pH regardless of the mechanical control of biofilm and disease (due to no relevant interaction). Thus, even at strict controlled oral hygiene, sucrose can disrupt the saliva pH, probably due to the large increase in salivary lactic acid (pKa = 3.87: completely dissociated at physiological pH) from the biofilm and salivary sediments^27^. This outcome can prove that the high rates of dietary carbohydrate consumption in both groups (represented here by more than 50% of the childhood diet) may promote acidogenic adaptation specialized in fast sugar metabolism. This hypothesis fits well with a previous investigation that showed a lower salivary pH in orally healthy individuals with the dominance of saccharolytic microbiota ecotypes, when compared with those adapted to proteolysis, even after an overnight fasting state^4^.

Despite the main effect of the disease was not observed in salivary pH, the spreading interaction between biofilm and disease (α = 0.073, β = 0.44, ηp^2^ = 0.06) indicates that the behavior of saliva pH at the pre- and post-rinse moments is codependent of these factors. Specifically, the mechanical control of biofilm had no impact on the saliva pH of children with ECC but caused a greater reduction in the saliva pH of CF children at the post-rinse moment. Concerning CF children, a plausible explanation for the lower post-sucrose pH is that good oral hygiene may provide a less-structured exopolysaccharide matrix with greater ability to diffuse H^+^ for saliva^28,29^. Regarding the children with ECC, authors believe that the dysbiotic state could favor a coercive environment for a saccharolytic metabolism even in the case of good oral hygiene. Thus, this framework suggests that regardless of caries disease, more important than the overall glycolytic metabolism per se would be the fighting between symbiotic and dysbiotic microbiome.

An important remark is that our findings only evaluated changes in the saliva’s pH five minutes after sucrose exposure. Therefore, since some studies reported that the biofilm pH profiles of caries-affected individuals have more prolonged pH falls than caries-free individuals^30–32^, the role of biofilm disruption in the shape of the Stephan curve should be considered when distinguishing CF children from those with ECC. In this context, screening the metabolic and microbial composition of saliva and its interactions with the biochemical process in biofilm structure can provide a more comprehensive understanding of this process.

The findings of our study show that, with the mechanical control of biofilm, the expected higher buffering capacity in CF children was compensated by an analogous response to sucrose exposure (no difference between groups was observed at the post-sucrose rinse moment). Conversely, the absence of mechanical control of biofilm (as a simulation of poor oral hygiene) approximated the buffering capacity of CF to children with ECC in the fasting state (no significant difference between them), while in the presence of a sucrose rinse, the buffering capacity of saliva in CF children was higher than in children with ECC. Taken together, these results can indicate that CF children appear to regulate more effectively the saliva pH than children with ECC and that aproper mechanical control of biofilm mediates the ability of children with ECC to resist the chemical aggression imposed by sucrose exposure. Thus, ECC and poor oral hygiene can be linked to an imbalance in the buffer capacity.

Regardless of the influence of ECC on buffering capacity, two further aspects are worth noting: firstly, the strategy of mechanical control of biofilm tended to reduce the BC after sucrose rinse. Secondly, the sucrose rinse did not induce changes in BC when the mechanical control of biofilm was absent. These outcomes strengthen the hypothesis that biofilm can mediate salivary functions due to its influence on the ionic and molecular transport to saliva. Noteworthy, we emphasize that the more organized extracellular polymeric matrix grounded by a heavy biofilm formation is critical for understanding the pH variations in the oral environment and the individual ability to buffer H + in saliva. In this regard, the tooth-biofilm-saliva interplay cannot be overlooked to avoid the misunderstanding of our data. The biofilm accumulation under frequent sucrose exposure provides a favorable environment for a more cariogenic microbiota and for the formation of an EPS-rich matrix, wherein an increase in the cellular glycolytic metabolism in fasting and feast conditions is highly expected. The enhancement of biofilm caries-inducing properties includes the biological sealing of biofilm structure, which also reduces the accessibility and ionic exchange. Therefore, the leaving of organic acids and the entering of bicarbonate to neutralize them becomes more difficult^28,29^. With this background, we argue that the lower buffer capacity in the mechanical control of biofilm situation suggests that more organic acids come from the biofilm and are diluted in the saliva in the same way that more bicarbonate interacts with biofilm.

Recognizing the role of CA VI in the bicarbonate pH-buffering system behavior^9–11^, we investigated the catalytic activity of CA VI under the experimental conditions tested in this study. Regarding the main effect of the disease, consistent with previous studies^12,14,17^, caries-active subjects had a higher CA VI activity when compared with caries-free subjects. Moreover, the nuances of buffering capacity according to healthy and diseased status imply that protein function is profoundly affected by ECC. Although the understanding of this biological behavior depends on a comprehensive study of the biochemical interaction in the oral environment, as well as on its impact on the enzymatic kinetic (out of the scope of this clinical study), the prolonged state of imbalance and the substantial genetic component are both plausible explanations. As a bioactive molecule, the cross-linking with charged components (e.g., molecules, ions, and oral bacteria) can cause chemical perturbations (maybe irreversible changes) that will affect deliberately the protein structure and function^33,34^. In support of the innate susceptibility hypothesis, CA VI gene polymorphisms have been associated with dental caries status^35^, buffering capacity^36^, and upregulation of colonization for cariogenic species as *S. mutans* and *S. wiggsiae*^37^.

The short-term mechanical control of biofilm strategy stabilized CA VI activity and thereby preserved the pre-sucrose rinse catalytic behavior in CF and ECC groups. Interestingly, when mechanical control of biofilm was absent, CA VI activity suffered a potential inhibitory effect mediated by sucrose. This effect was more pronounced in children with ECC, whose enzyme activity significantly dropped down to only 70% of its initial activity after sucrose rinse. Since saccharolytic bacteria have a complex metabolic pathway—Embden-Meyerhof-Parnas pathway and its branches—ruled by changes in the sugar supply^38^, we argued that poor oral hygiene causes an increase in metabolites profile, after the transient sucrose exposure, that would be so high that it would change the CA VI catalytic activity. Furthermore, the recognition of the mechanical control of biofilm as a strategy of bacteria removal and oxidative stress decreasing^39^ it is remarkably important to explain the distinguished behavior of CA VI according to biofilm intervention since the possibility of protein chemical shift perturbation is reduced.

The reduction in CA VI activity after sucrose in this simulation of poor oral health strengthens our hypothesis that changes in the catalytic site of the carbonic anhydrase VI could be expected due to a shift toward a dysbiotic (or at least unhealthy) state. Accordingly, the most exciting discussion here is if the higher CA VI activity in children with ECC (even in fasting situations) is an adaptation (upregulation) to a more intense chemical aggression due to the sucrose-enriched diet and poor oral hygiene practices. Could it be possible that the nature of this enzymatic over-specialization leads to side effects as an impaired acid–base control in children with ECC, thereby making these subjects less resistant to the cariogenic challenge? For this purpose, the determination of causality can be elusive.

Under the experimental conditions of our study, although no factor (sucrose rinse, biofilm, or disease) individually influenced the α-amylase activity, the significant interaction between these factors (α = 0.043, β = 0.53, ηp^2^ = 0.07) indicates a complex mechanism. An in silico study about amylase interactome identified several amylase-forming cross-linking that could be related to important roles towards oral health maintenance^19^. However, regarding the relationship between α-amylase and its effects on the neutralization of chemical aggression through CA VI or its influence on pH and buffering capacity, we could not explain these particular behaviors.

The lack of information regarding salivary metabolic profile, composition and thickness of biofilm, microbiological dominance, and sucrose clearance put some restrictions in our findings. The cariogenic challenge with a 20% sucrose solution gives limited information concerning the clinical reality since the severity of the cariogenic potential of sucrose also depends on their physical form and retentiveness^40^. Concerning CA VI activity, despite the *in-gel* zymography method being a powerful strategy to analyze enzyme activity^41^, this observation used the optimization of reaction conditions to simply imply that protein function is somehow affected. Thus, we must highlight that this research did not evaluate the intrinsic aspects of the CA VI structural changes and their specific impact on the catalytic efficiency of this iso-enzyme.

To avoid misunderstanding our data, one must bear in mind that, despite the differences in saliva pH according to the experimental condition, no saliva sample showed variation below to the pKa = 6.1 of the bicarbonate buffer (when the effectiveness of this buffer reduces)^8^. This is a reason to believe that the buffer effect can be particularly different in the biofilm structure, in which the EPS-rich biofilm matrix creates spatially heterogeneous acidic microenvironments with state-stable pH under the critical demineralization point for enamel (pH < 5.5)^29^. Moreover, pH changes to values lower than the CA VI isoelectric point (pK ≅ 6.0) can lead to conformational changes in the CA VI structure and function due to their zinc-bound water electrostatic interactions^34^.

Another important cautionary note is that the interaction effect for some dependent variables was impacted by low power. To perform this study, the sample size was estimated considering the CA VI activity (variable with the greatest variability), an α-value of 0.05, and an β-value of 0.15. However, the statistical planning to analyze data of the study (three-way mixed model analysis of variance) did not match with the sample size calculation (the difference between two groups). It must be considered that as this was the first study designed with this objective, we did not have any preliminary data to precisely assess the expected variability and thus determine the appropriated power calculations. In this context, we provide statistical results in a thorough, honest, and useful way to ensure the conduction of new and independent experiments to increase the quality of the evidence in this research area.

In conclusion, mechanical control of biofilm did not modify the ability of saliva to neutralize the oral environment after sucrose exposure in children with ECC. On the other hand, CF children appeared to regulate more effectively the saliva pH than those with ECC, while the absence of mechanical control of biofilm mediates their pH-modifying ability after sucrose exposure.

## Methods

### Subjects

Sample size calculation was done using the Gpower 3.1 software, considering the assumptions of parametric statistics and an α-value of 0.05, β-value of 0.15, allocation rate of 1/1, and a confidence interval of 0.95. The mean (standard deviation) of the CA VI activity in saliva before sucrose rinse—caries-free = 19.13 (16.91), and ECC = 42.75 (32.47)—was used as a basis for the calculation^15^. The sample size was 23 volunteers in each group, but this value was increased by 20% (n = 28) to compensate for possible losses during the phases of the study.

The sampling procedure considered the use of a probabilistic single-clustering method. Once the public daycare centers in Piracicaba (São Paulo, Brazil) were selected, all children were screened and included in the study according to the eligibility criteria. The inclusion criteria were children of both sexes, aged 4 to 5 years old, and with caries disease (diagnostic thresholds including all visible signs of caries that can be assessed by the decayed, missing, and fiiling surfaces (dmfs) plus the diagnosis of initial active caries lesions) or no caries (absence of caries experience and initial active caries lesions).The exclusion criteria considered any systemic disease, neuromotor or communication difficulties, severe fluorosis, altered salivary flow (below 0.7 mL), use of orthodontic appliances, antibiotic therapy, and also those preschoolers whose parents or guardians refused to participate in the research or who did not cooperate with the clinical examinations. The objective of the eligibility criteria was to reduce the influence of factors that could change the composition and metabolism of microorganisms (use of antibiotics), that could affect the saliva flow rate and composition (systemic diseases), increase the risk and severity of dental caries (systemic diseases, dental fluorosis, and use of orthodontic appliances), or prejudice the compliance with the protocol recommendations (neuromotor or communication difficulties). This information was obtained from a questionnaire filled out by parents. The questionnaire was based on Bhattarai et al.^42^ and adapted to this research.

The recruitment procedure was achieved only when the number of confirmed eligible children in each group corresponded to the required sample size. Children who were identified with urgent treatment needs were referred to the Pediatric Clinic of the Piracicaba Dental School—University of Campinas, where they received full oral care.

### Experimental design

A quasi-experimental study was carried out in a sample composed of caries-free (CF) children and children with ECC to investigate the codependent impact of two experimental conditions: the mechanical control of biofilm and a cariogenic challenge with a 20% sucrose rinse.

The situation of proper oral hygiene (mechanical control of biofilm situation) and poor oral hygiene (no mechanical control of biofilm) was carried out in different moments within the time–space of at least one week. These interventions were conducted as follows:

- Mechanical control of biofilm situation: for two days, the volunteers were submitted to mechanical control at least three times a day with a proper technique (Fones technique). Toothbrushing was performed 1 h prior to saliva collection and supervised by the first author who carried out this procedure always in the morning (between 8:00 and 9:00 a.m.), and in the afternoon (between 4:00 and 5:00 p.m.). Phone calls and/or text messengers were used as a strategy to remind parents regarding the nighttime toothbrush.
- No mechanical control of biofilm situation: for 2 days, parents were advised through phone calls and/or text messengers that their children should abstain from toothbrushing. To strength this recommendation, children were also instructed regarding the procedure. We also verified visible biofilm on the upper jaw incisors^43^ as a control for toothbrushing abstention.

**Figure 6:**
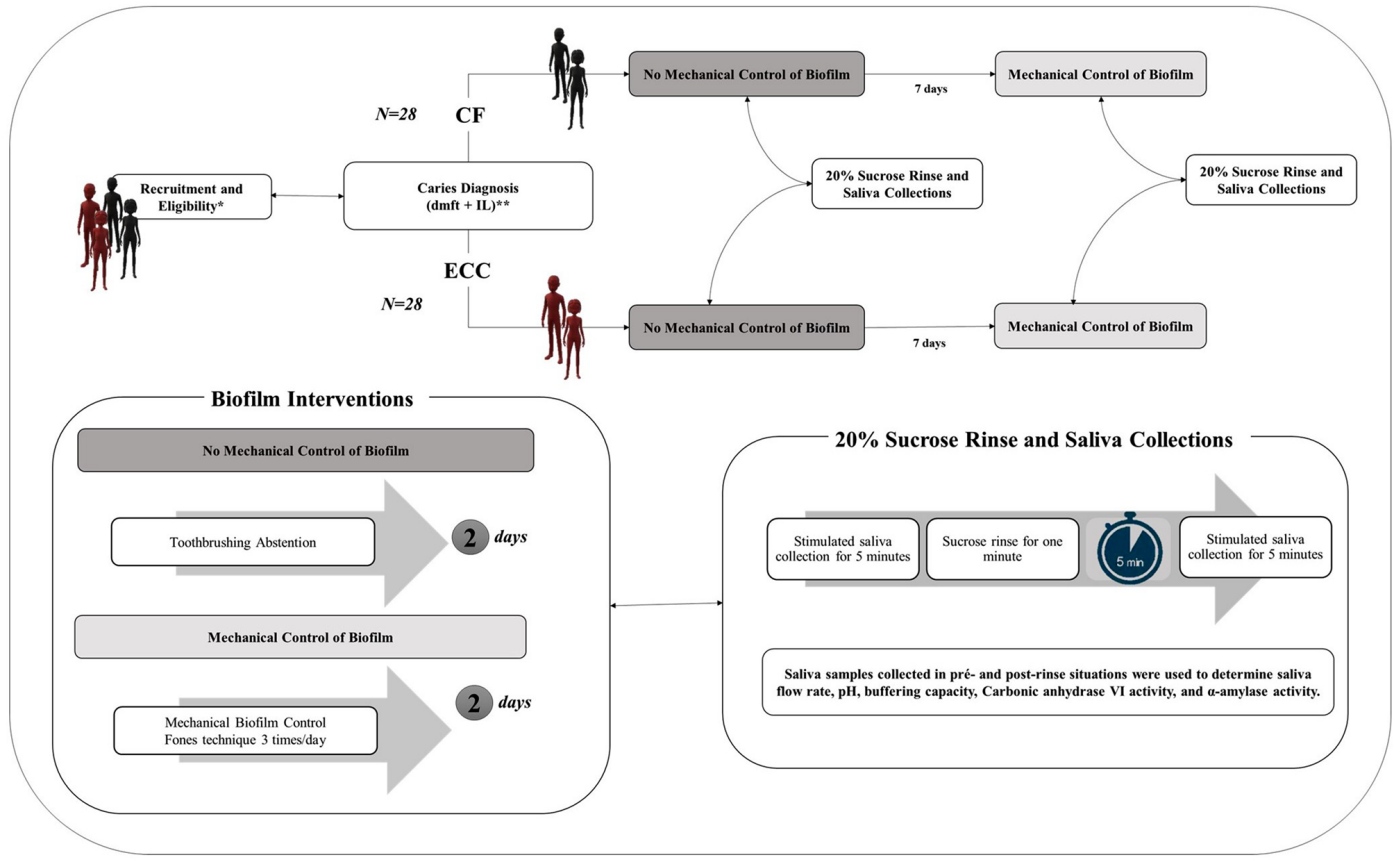
Scheme of the experimental design. During the study, we collected information regarding sucrose intake. * Recruitment and Eligibility: Potentially eligible children who were invited to participate (n = 250) and 170 were excluded because parents refuse to sign the informed consent (n = 128) or the number of confirmed eligible children in each group was completed (n = 42) ⟶ 80 Children were examined for eligibility, from those 24 were excluded because they refused to cooperate (n = 6), had a systemic disease (n = 2), had communication difficulties (n = 1), their parents did not cooperate (n = 10) or the number of confirmed children in their respective group was completed (n = 5). The number of confirmed eligible children were 56 according to the equal allocation in the CF and ECC group. **Caries Diagnosis: Caries diagnosis of each dental surface (dmfs) according to the recommendations by the World Health Organization^41^, along with the diagnosis of initial active caries lesions (IL)^43^. CF = Caries-free: Absence of caries experience and initial active caries lesions. ECC = Early Childhood Caries: Diagnostic thresholds including all visible signs of caries that can be assessed by dmfs plus diagnosis for initial active caries lesions.

In each situation, we collected saliva samples before and five minutes after a rinse during 1 min with 10 mL of a 20% sucrose solution. The collected saliva samples were used to determine flow rate, pH, buffering capacity, CA VI activity, and α-amylase activity. Information about the child’s dietary habits was also collected during the period of the experimental procedures. Figure 6 shows a full representation of the experimental design. Sucrose was used in the concentration of 20% due to the similarity with some infant foods and because it simulates the use of ≈ 2 soup spoon of sugar in 100 mL of solution.

### Training of the examiner and dental caries assessment

To ensure the validity of the dental caries assessment, the examiner was submitted to a coaching exercise that involved theoretical and clinical phases. The first step comprised a theoretical discussion of the individualized caries diagnosis of each dental surface (dmfs) according to the recommendations by the World Health Organization^44^, along with the diagnosis of initial active caries lesions (IL), as an adaptation of Nyvad criteria^45^. The WHO and IL criteria and codes were adopted following the protocol designed by Assaf et al.^46^. Initial active caries lesion was defined as an enamel lesion with abnormal rough, increased opacity or whitish/yellowish discoloration, and loss of luster. To be recorded, the enamel surface should be free of clinically detectable loss of dental tissue or have localized surface defects (microcavity) only in enamel.

The second step evaluated the analysis consistency in a population of the same age as the sample. A pediatric dentist and a calibrated examiner (gold standard) assessed the caries index in 10 children selected at random from the sample. Inter-rater reliability was checked using Cohen's kappa statistics (κ = 0.89), and the reproducibility of the diagnosis was determined by the intra-examiner from reevaluations in 60% of children after a one-week interval (κ = 0.95).

After the training exercise, the calibrated examiner carried out a clinical test at the daycare center. During the examination of each child, he used sterilized clinical material (a clinical mirror, a blunt-ended explorer) for each child as well as personal protective equipment. A portable LED flashlight (SFL5540, Philips, Brazil), a portable triple syringe (Odonto case Baseline, Rio de Janeiro, Brazil), and sterilized gauzes (Medi House, Sao Paulo-SP, Brazil) were also used to facilitate the diagnostic of the ACL.

### Analysis of dietary habits

The approach used considered the five steps of the US Department of Agriculture Automated Multiple-Pass Method for collecting dietary recalls^47^. Parents or guardians filled out a three-day diet diary. The diary specified the children's eating habits, including the time of feeding and the amount and content of all meals and snacks. A detailed review of all food items and meals throughout the day was made to check for errors or omissions, and we collected more detailed information during an interview with the children’s parents or guardians (food type, brands, food preparation, bottle use, bottle preparation, and nighttime breastfeeding). Moreover, considering that all children included in this research stayed full-time at the daycare center (between 7:30 a.m. and 5.00 p.m.), the content of the institutional menu was gathered and the information was added to the analysis.

The frequency of sugar exposure in each day was counted and then divided by the number of days (three). A blinded researcher (nutritionist) analyzed this dietary information and, with the help of software, assessed the amount of fermentable carbohydrates ingested by each child. The amount of sugar ingested per day (grams/day) and the relative percentage of sugar amount concerning all macronutrients from the diet were estimated by the average consumption in grams in the three days of the dairy filling^14^.

### Saliva collections

Collections were performed at least one and a half hours (1:30 h) after the last meal or toothbrushing. The same researcher collected the stimulated saliva samples from each child always at the same time of the day (between 9:30 a.m. and 11:00 a.m.). This time was considered to reduce the influence of circadian variations on the parameters evaluated and to ensure that CA VI and α-amylase, both with parallel variation during the day, would be present in the oral cavity at their highest concentrations^48^. To stimulate the saliva secretion, we used a piece of Parafilm M (Pechiney Plastic Packaging, Inc. Manufacturing and Markets Plastic, Chicago, Illinois, USA), as previously described by Dawes and Kubieniec^49^. The initial saliva (produced in the initial 30 s) was put away, and the rest was collected during 5 min in autoclaved graduated tubes. After the material was collected, we calculated pH and buffering capacity, and stored the graduated tubes in a cooler during the transport to the laboratory, where we analyzed the saliva flow rate. The saliva samples were centrifuged at 16,097.2 g for 15 min and then transferred for identified microtubes. Samples were kept frozen at − 40 °C until the analyses of total protein, and activity of CA VI and α-amylase. The concentration of total protein was assessed by Bradford colorimetric method^50^; and used to normalize the activity of CA VI and α-amylase of each sample.

### Determination of saliva flow rate, pH, and buffering capacity

The saliva flow rate was measured considering the volume collected divided by the collection period (mL/min)^51^. The pH was estimated in 0.3 mL of saliva with a pH Orion microelectrode (ThermoScientific, Waltham, MA, USA) precalibrated with standard solutions with pH 4 and 7. To evaluate saliva buffering capacity, 3 µL increments of 0.25 M HCl were added to 0.3 mL of saliva in a microtube that was shaken (pH was determined until pH 4 was reached). For the buffering capacity, we used the following equation: BC = ΔC/ΔpH, where ΔC is the total amount of HCl used to reduce the initial pH to pH 4.0, and ΔpH the change in salivary pH. The buffering capacity and saliva pH were evaluated in the first 30 min after the collection and, during this period, the samples remained in a closed tube to avoid CO_2_ loss^8^.

### Determination of CA VI activity

Carbonic anhydrase VI activity was determined using the zymography method^52^ modified by Aidar et al.^53^. The principle of this method was based on the use of polyacrylamide gel electrophoresis (PAGE) to identify the CA VI activity by immersing the gel in CO_2_-saturated deionized-water. After the electrophoresis running to trap the CA isoenzymes, the gel was stained with a pH indicator (bromothymol blue). The blue background corresponds to the alkaline pH of PAGE gel (near to 8.2) and the yellow band corresponds to the CA VI isoenzyme that reacts with CO_2_ of the medium to drop of pH until a chemical equilibrium is achieved (acidic pH).

In this analysis, 30 µL of collected saliva were mixed to 30 µL of electrophoresis buffer, and then 10 µL of this material were pipetted into each channel of 13.5% of a PAGE. The PAGE was placed in an electrophoresis buffer with suitable electrolytes, and a voltage of 140 V was applied for 1 h and 50 min, which caused a migration of negatively charged molecules through the gel in the direction of the positively charged anode. After that, the gel was incubated in 0.2% bromothymol blue for 10 min. The reaction of the CA VI was observed by immersing the gel in purified water saturated with CO_2_ (dry ice). The gel was photographed, and the images were analyzed using the Image J software, which transformed the bright areas of the CA VI bands into a numerical value. These values were normalized by total protein concentration, and CA VI activity was expressed as pixel/µg of protein.

### Determination of α-amylase activity

The salivary α-amylase activity was determined to utilize a kinetic enzyme assay kit, specifically designed and validated for the kinetic measurement of salivary α-amylase activity (Salimetrics, State College, PA, USA). This method uses a chromogenic substrate (2-chloro-p-nitrophenol linked with maltotriose) that is converted by salivary α-amylase to a product (2-chloro-p-nitrophenol) that can be spectrophotometrically measured^54^. The absorbance of each 96-well plate was photometrically quantified using an ELISA plate reader at 405 nm wavelength (EPOCH, BioTek Instruments, Inc., Winooski, VT, USA). The increase in absorbance is directly proportional to the increase in α-amylase activity. We calculated these values based on the standardization of the kit curve and according to the manufacturer's instructions. The activity of α-amylase was expressed in unit activity per milligram of total protein (U/mg).

### Statistical approach

For statistical inferences, we used the SPSS software for Windows, version 21.0 (SPSS, Inc., Chicago, IL, USA) and the GraphPad Prism 7.04 software (GraphPad Software, La Jolla, USA). The data were checked for normality and homogeneity with the Shapiro–Wilk and the Levene tests, respectively. The values of pH and CA VI activity fitted the normal distribution and variances homogeneity, whereas SFR and BC were transformed with the logarithm of 10, and α-AML with the squared root. The equality of multiple variance–covariance matrices was proven using Box’s M test.

Differences between groups regarding sex and sugar diet intake were assessed using independent t-test and chi-squared. A three-way mixed model analysis of variance was used to evaluate the interaction between the two within-subjects factors (environment: mechanical control of biofilm or no mechanical control of biofilm, and rinse: pre- and post-rinse), and one between-subjects factor (disease: ECC and CF) in saliva variables (flow rate, pH, buffering capacity, CA VI activity, and α-AML activity). The simple effects test was carried out as a single-step pairwise comparison procedure considering the Bonferroni adjustment applied for multiple comparisons. A significance level of 0.05 was established for the analyses.

### Ethics declarations

#### Approval for human experiments

Methods were carried out following the Helsinki declaration and the regulatory guidelines and norms obey the 466/12 resolution for research ethics in Brazil. The Research Ethics Committee of the Piracicaba Dental School University of Campinas approved this research (CAAE: 70777517.9.0000.5418). Parents or guardians who agreed to include their children in this study signed informed consent, authorizing their participation.

## Supplementary Information

Supplementary Information.
