# Supplementary material for: The role of mechanical control of biofilm in the salivary pH after sucrose exposure in children with early childhood caries
Source: Sci Rep. 2021 Apr 5;11:7496. doi: 10.1038/s41598-021-86861-4 (PMC8021547; doi:10.1038/s41598-021-86861-4)
Supplement: Supplementary file 1 — Supplementary Information. [file 41598_2021_86861_MOESM1_ESM.docx]

**The role of mechanical control of biofilm in the salivary pH after sucrose exposure in children with early childhood caries**

Aline Tavares Lima-Holanda^1^, Emerson Tavares de Sousa^1^, Marinês Nobre-dos-Santos^1*^, Carolina Steiner-Oliveira^1^.

^1^Department of Pediatric Dentistry, Piracicaba Dental School, University of Campinas-UNICAMP, Limeira Avenue 901, Piracicaba-SP, Brazil, CEP 13414-903.

**Supplementary Material**

| Biofilm Intervention | Disease | SFR | | pH | | BC | |
| --- | --- | --- | --- | --- | --- | --- | --- |
|  |  | *Pre-rinse* | *Post-rinse* | *Pre-rinse* | *Post-rinse* | *Pre-rinse* | *Post-rinse* |
|  |  | *Median (IQR)* | *Median (IQR)* | *Mean (SD)* | *Mean (SD)* | *Median (IQR)* | *Median (IQR)* |
| *Biofilm Control* | *CF* | *1.11 (0.59)aA* | *1.33 (0.85)aB* | *7.58 (0.32)aA* | *7.21 (0.39)aB** | *1.40 (0.48)aA* | *1.22 (0.33)aB** |
|  | *ECC* | *1.21 (0.84)aA** | *1.41 (1.09)aB** | *7.61 (0.33)aA* | *7.39 (0.38)aB* | *1.24 (0.42)bA* | *1.16 (0.46)aB** |
| *No Biofilm Control* | *CF* | *1.16 (0.50)aA* | *1.41 (0.61)aB* | *7.70 (0.34)aA* | *7.48 (0.39)aB** | *1.55 (0.57)aA* | *1.42 (0.37)aA** |
|  | *ECC* | *1.04 (0.43)aA** | *1.12 (0.53)aA** | *7.66 (0.29)aA* | *7.37 (0.27)aB* | *1.39 (0.43)aA* | *1.26 (0.45)bA** |
| *Three-way Mixed Model* | | *p-value (Power)* | ηp² | *p-value (Power)* | ηp² | *p-value (Power)* | ηp² |
| *Rinse* | | *0.00 (1.00)* | *0.411* | *0.000 (1.00)* | *0.619* | *0.000 (0.99)* | *0.28* |
| *Biofilm* | | *0.014 (0.70)* | *0.107* | *0.051 (0.50)* | *0.069* | *0.006 (0.81)* | *0.13* |
| *Disease* | | *0.78 (0.06)* | *0.001* | *0.763 (0.06)* | *0.002* | *0.002 (0.88)* | *0.16* |
| *Rinse *Biofilm* | | *0.46 (0.11)* | *0.010* | *0.581 (0.085)* | *0.006* | *0.397 (0.13)* | *0.013* |
| *Rinse*Disease* | | *0.71 (0.07)* | *0.003* | *0.561 (0.089)* | *0.006* | *0.816 (0.06)* | *0.001* |
| *Biofilm*Disease* | | *0.048 (0.511)* | *0.071* | *0.073 (0.435)* | *0.058* | *0.928 (0.05)* | *0.000* |
| *Rinse*disease*Biofilm* | | *0.42 (0.13)* | *0.012* | *0.123 (0.34)* | *0.043* | *0.696 (0.07)* | *0.003* |
| *Box’ M: p-value* | | *0.310* | | *0.741* | | *0.174* | |

**Evaluation of the interaction between rinse, biofilm intervention, and disease on the salivary flow rate (SFR), pH, and buffering capacity (BC): a Three-way Mixed Model.**

*Statistical analyses were performed with a sample of 56 volunteers, 28 per group.* ηp²: *Partial eta squared. Gaussian-distributed data were expressed in mean followed by the standard deviation. Non-Gaussian-distributed data were expressed in the median followed by the interquartile range. To fulfill the ANOVA premises, SFR and Buffering Capacity data were transformed by taking the logarithm of 10. Different small letters represent statistically significant simple effects for disease factors (CF versus ECC) within each environmental factor. Different capital letters represent statistically significant simple effects for rinse (pre- versus post-rinse). Asterisks expressed simple effects for biofilm factor (biofilm absence versus biofilm presence) within CF and ECC children.*

**Evaluation of the interaction between rinse, biofilm intervention, and disease on CA VI activity and α-AML activity: a Three-way Mixed Model.**

| Biofilm Intervention | Disease | CA VI | | α-AML | |
| --- | --- | --- | --- | --- | --- |
|  |  | *Pre-rinse* | *Post-rinse* | *Pre-rinse* | *Post-rinse* |
|  |  | *Mean (SD)* | *Mean (SD)* | *Median (IQR)* | *Median (IQR)* |
| *Biofilm Control* | *CF* | *13.40 (7.96)aA* | *16.04 (8.37)aA** | *12.55 (7.84)aA** | *14.73 (10.75)aA* |
|  | *ECC* | *23.21 (9.28)bA* | *21.76 (10.50)bA** | *16.66 (9.41)aA* | *14.29 (8.66)aA** |
| *No Biofilm Control* | *CF* | *13.52 (8.89)aA* | *10.12 (5.99)aA** | *16.82 (7.73)aA** | *16.63 (6.23)aA* |
|  | *ECC* | *23.02 (10.46)bA* | *16.00 (6.32)bB** | *13.91 (8.86)aA* | *15.64 (8.32)aA** |
| *Three-way Mixed Model* | | *p-value (Power)* | ηp² | *p-value (Power)* | ηp² |
| *Rinse* | | *0.014 (0.705)* | *0.107* | *0.401 (0.13)* | *0.013* |
| *Biofilm* | | *0.013 (0.714)* | *0.109* | *0.029 (0.60)* | *0.085* |
| *Disease* | | *0.000 (0.998)* | *0.316* | *0.952 (0.05)* | *0.000* |
| *Rinse *Biofilm* | | *0.002 (0.88)* | *0.157* | *0.152 (0.30)* | *0.038* |
| *Rinse*Disease* | | *0.038 (0.553)* | *0.078* | *0.846 (0.05)* | *0.001* |
| *Biofilm*Disease* | | *0.973 (0.05)* | *0.000* | *0.26 (0.20)* | *0.023* |
| *Rinse*Biofilm*Disease* | | *0.90 (0.05)* | *0.000* | *0.043 (0.53)* | *0.073* |
| *Box’ M: p-value* | | *0.873* | | *0.432* | |

*Statistical analyses were performed with a sample of 56 volunteers, 28 per group.* ηp²: *Partial eta squared. Gaussian-distributed data were expressed in mean followed by the standard deviation. Non-Gaussian-distributed data were expressed in the median followed by the interquartile range. To fulfill the ANOVA premises, α-AML was transformed by taking the square root.*

*Different small letters represent statistically significant simple effects for disease factors (CF versus ECC) within each environmental factor. Different capital letters represent statistically significant simple effects for rinse (pre- versus post-rinse). Asterisks expressed simple effects for biofilm factor (biofilm absence versus biofilm presence) within CF and ECC children.*
